# Strategic dam site selection and hazard mapping using remote sensing: insights from Wadi Araba, Egypt

**DOI:** 10.1038/s41598-026-39883-9

**Published:** 2026-03-19

**Authors:** Mona A. Mesallam, Zenhom E. Salem, Ayman M. Al Temamy, Tamer S. Abu-Alam, Amr S. Fahil

**Affiliations:** 1 Geology Department, Faculty of Science, Tanta University, Tanta, 31527 Egypt; 2 Geophysical Exploration Department, Desert Research Center, El Matariya, Egypt; 3 Department of Arctic Marine Biology, UiT The Arctic University of Norway, Tromsø, Norway; 4 Outermost Regions Sustainable Ecosystem for Entrepreneurship and Innovation (OSEAN), University of Madeira Colégio dos Jesuítas, Funchal, Portugal

**Keywords:** Remote sensing data, GIS modelling, North africa, Dam potential, Flash floods, Geographic information systems, Environmental sciences, Hydrology, Natural hazards

## Abstract

Flash floods represent one of the most recurrent and devastating geohazards affecting Egypt’s northern area of the Red Sea coast, particularly in hyper-arid catchments such as Wadi Araba. This study develops a geospatial decision-support framework to identify flash-flood-prone areas and determine optimal dam locations for flood mitigation and groundwater recharge. The analysis incorporates eight thematic layers: slope, LULC, rainfall, DEM, drainage density, lineament density, distance to main streams and roads. The data used for thematic layers was derived from satellite imagery and ancillary datasets, processed using remote sensing and GIS tools. A multi-criteria decision analysis (AHP) generated a weighted overlay model of flood susceptibility. The resulting map classified Wadi Araba into high-risk (0.53 km²), moderate-risk (2354.8 km²), and low-risk (1671.34 km²) zones, with the highest risk concentrated in the southern Galala Plateau, moderate-risk zones occurring in lowland and downstream basins, and low-risk zones mainly located in the northern Galala Plateau and western Wadi Araba. The RS–GIS/AHP framework identified top-ranked dam sites with favourable storage geometry; validation returned medium AUC for susceptibility of flash flooding (≈ 0.6–0.7) and good AUC for dam suitability (≈ 0.7–0.8).

## Introduction

Floods remain one of the most recurrent and destructive natural hazards worldwide, threatening lives, infrastructure, and economic stability. In hyper-arid and semi-arid regions, flash floods are particularly devastating because they occur suddenly and interact with fragile infrastructure and scarce water resources. Effective flood management, therefore, requires robust, reproducible frameworks that can identify vulnerable areas and guide the planning of protective structures, including dams that serve both disaster-risk reduction and water-storage functions^1–6^. Developing such frameworks is especially critical in data-scarce regions, where conventional hydrological models are difficult to calibrate and where climate variability further complicates long-term planning.

Recent advances in geospatial and decision-support tools provide a powerful avenue for addressing this challenge. The integration of remote sensing (RS) and geographic information systems (GIS) with multi-criteria decision-making (MCDM) techniques, particularly the Analytical Hierarchy Process (AHP), has proven effective in water resource management and hazard mitigation^7–13^. These approaches enable the systematic combination of diverse environmental and infrastructural factors, incorporate expert knowledge, and include consistency checks to ensure reliability. Moreover, coupling such frameworks with machine learning and artificial intelligence can improve forecasting and enhance the accuracy of site selection for hydraulic infrastructure^8,10^.

**Fig. 1:**
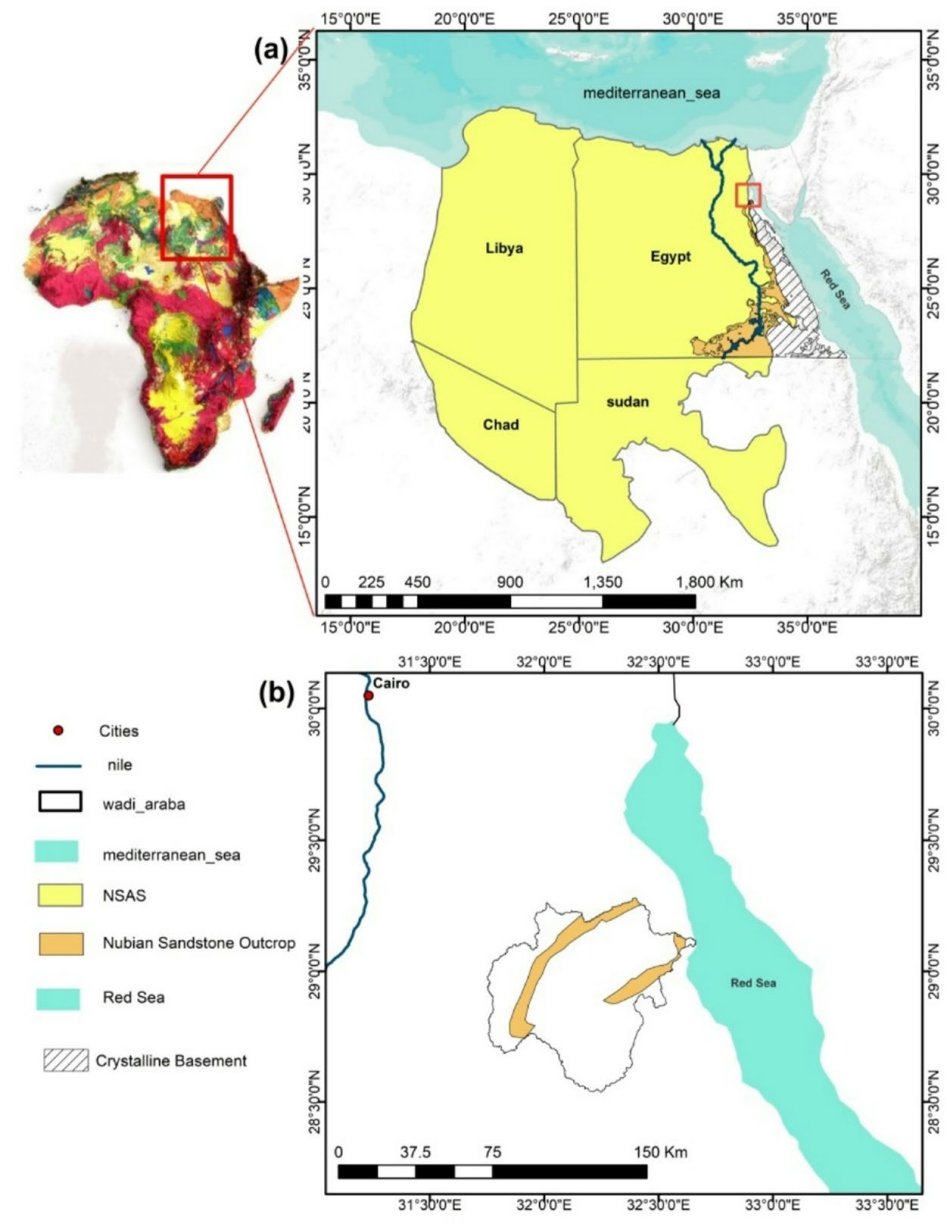
Wadi Araba location, Eastern Desert, Egypt. (**a**) Regional map showing Egypt at North africa, crystalline basement, Wadi Araba extent, and Nubian Aquifer System. (**b**) close up of the Wadi Araba watershed showing its extent and main geographic features. (generated in ArcGIS 10.8 (https://www.esri.com/en-us/arcgis/products/arcgis-desktop/overview*))*.

Wadi Araba, located along the western margin of the Gulf of Suez between the Northern and Southern Galala Plateaus (Fig. 1), Egypt, provides a critical test case for such a methodology. The area is characterized by a hyper-arid climate, episodic flash floods, and complex geological and hydrological settings^2–6,14–18^. At the same time, it is undergoing rapid urban expansion through projects such as New Galala City, which intensifies pressure on water resources and increases exposure to flood hazards. Despite its importance, the region remains understudied, with limited hydrological records and few detailed assessments of flood susceptibility and water management potential.

This study develops and applies an integrated RS–GIS–AHP framework tailored to the characteristics of Wadi Araba. The approach generates flood susceptibility maps, validates them with ROC/AUC metrics, and identifies optimal sites for dam construction. The outcomes provide critical insights into both local and global contexts. Nationally, the results offer guidance for protecting infrastructure, reducing flood risk, and securing freshwater resources through managed aquifer recharge. Internationally, the study contributes to advancing reproducible methods for flood-hazard assessment in hyper-arid settings, while supporting the achievement of several Sustainable Development Goals (SDGs), particularly those related to clean water (SDG 6), resilient infrastructure (SDG 9), and climate adaptation (SDG 13).

This research builds upon the findings of our recently published study, which delineated groundwater potential zones in Wadi Araba by integrated RS–GIS–AHP approaches, representing a logical continuation of that work. The previous paper focused on delineating the hydrogeological framework and identifying regions with promising groundwater potential within the structurally challenging basin, whereas the present study redirects attention to surface hydrology by assessing flood susceptibility and prioritizing ideal locations for dam construction. This subsequent study connects groundwater potential with flood-hazard dynamics, yielding a integrated perspective of water-resource behavior in hyper-arid regions and presenting a practical framework for integrated water management, hazard mitigation, and sustainable development planning in Wadi Araba.

## Study area and settings

### Location

Wadi Araba is located along the western margin of the Gulf of Suez, between the Northern and Southern Galala Plateaus in Egypt’s Eastern Desert. The basin covers an area of about 2,800 km² (Fig. 1)^15,16^. Its ephemeral drainage network flows eastward across stepped escarpments and sandstone benches before dispersing over gravel-floored channels and a narrow coastal plain, where composite alluvial fans coalesce along basin margins^13,15,17^.

### Geological and tectonic setting

The bedrock consists mainly of Carboniferous–Lower Cretaceous Nubian Sandstone, overlain by Upper Cretaceous carbonate-platform successions of the Galala region. These units are partly covered by Quaternary alluvium and aeolian sand^18^. The structural framework reflects multiple tectonic phases: Late Cretaceous Syrian Arc shortening, expressed as E–W to ENE–WSW folds, was later overprinted by Oligo–Miocene rifting of the Gulf of Suez. This rifting generated NW–SE and NNE–SSW normal faults and tilted blocks that segmented the basin and influenced channel alignments^16,17^. Stratigraphy thickens toward the east, while the western hinterland is characterized by thinner successions and broader interfluves^15,16^.

### Hydrogeology and flood history

Groundwater resources are mainly stored in the Nubian Sandstone aquifer, where intergranular porosity is locally enhanced by fractures, and in fractured Upper Cretaceous carbonates. Aquifer thickness generally increases eastward toward the rift margin^18^. Despite the hyper-arid climate, short and intense winter storms often exceed infiltration capacity, generating rapid runoff and flash floods that pose significant risks to settlements and transport corridors along the Red Sea coastal plain. The flash flood at Ras Gharib on 26–27 October 2016 is a recent event that illustrates the vulnerability of the region^19,20^.

## Materials and methods

**Fig. 2:**
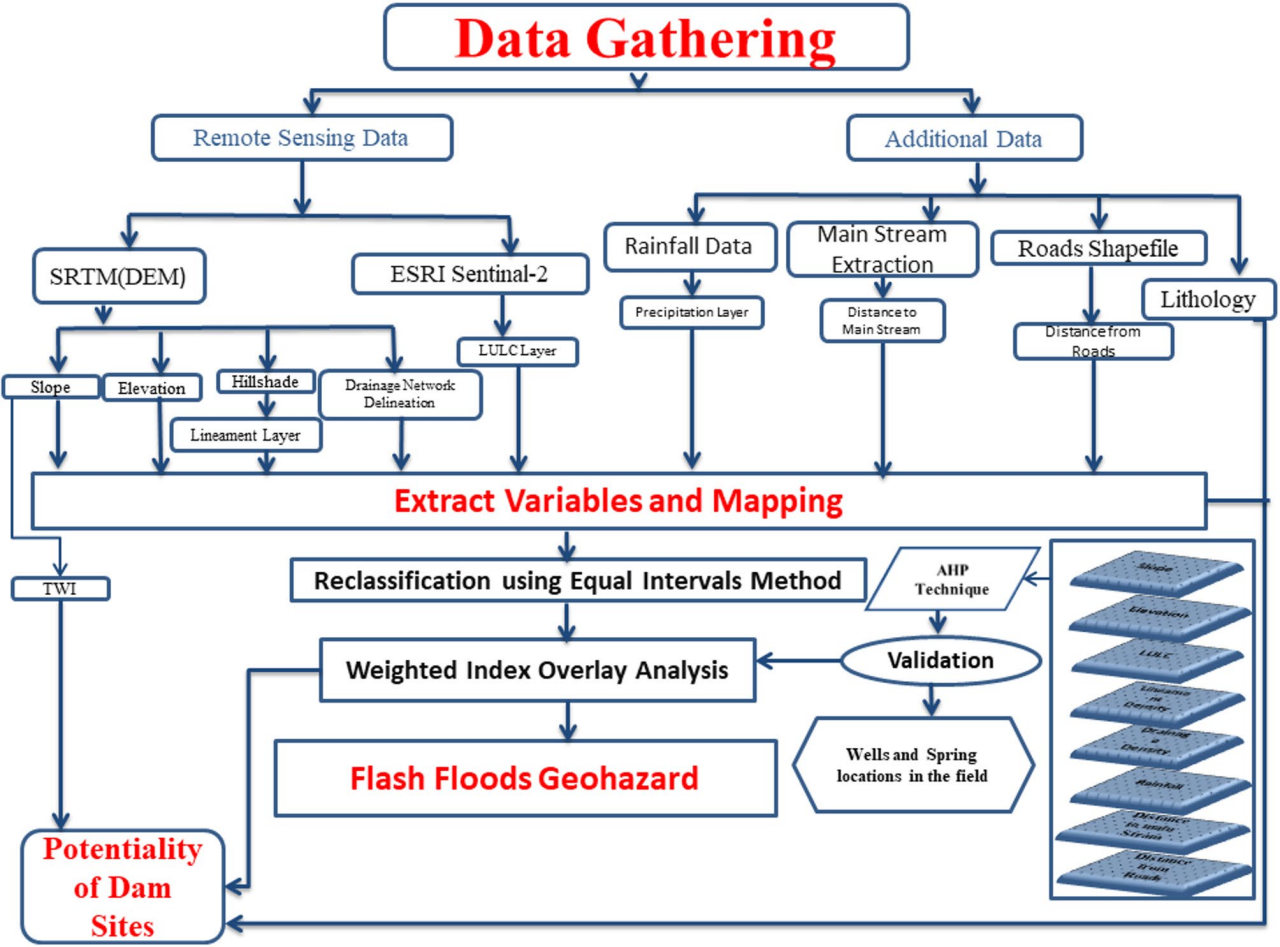
Workflow includes data gathering and variable derivation, five class reclassification, AHP weighting (CR ≤ 0.1), weighted overlay scoring, and ROC/AUC validation for flood susceptibility and dam siting results.

A reproducible RS–GIS workflow was implemented to extract flood susceptibility and dam siting factors from open datasets and supplementary vectors, adhering to Nature Portfolio reporting guidelines for methodological transparency and data availability. Primary sources included the SRTM 30 m DEM (https://earthexplorer.usgs.gov/), Sentinel-2-derived 10 m land-use/land-cover (https://livingatlas.arcgis.com/landcoverexplorer), a satellite precipitation product compiled into annual totals for 2011–2021, drainage/main-stream networks derived from the DEM, paved-road vectors, and regional lithology. All rasters were projected to WGS84/UTM zone 36 N, resampled to a uniform analysis grid, and preprocessed to extract slope, elevation, hillshade, lineaments, drainage density, distance to main streams, distance to roads, land use/land cover (LULC), rainfall, and the topographic wetness index (TWI) for multi-criteria analysis (refer to Figs. 2). Reclassification was conducted into five categories based on specific criteria, followed by the acquisition of Analytic Hierarchy Process (AHP) weights from pairwise comparison matrices, which were deemed acceptable only when the consistency ratio met the condition CR ≤ 0.1 prior to weighted-overlay scoring. Validation was performed utilizing independent evidence of flood impacts and hydrologically significant positives/negatives beyond the training set, with ROC/AUC calculated on a held-out subset and bootstrap confidence intervals specified in the Validation section.

### Remote sensing data sets

The satellite datasets and study area are consistent with our previously published work^14^; however, all analyses and figures in the present study were newly generated. Different data from satellite and optical images have been acquired to characterise the places susceptible to flash flooding and dam sites along the Wadi Araba basin.The SRTM DEM data was used to map the topographic features and determine the area of influence variables. Optical images were additionally utilized for assessing the land use land cover (LULC) of the research area. The satellite data, optical images, DEM and rainfull data were processed to build a dataset of 11 variables (slope, Landuse-Landcover, density of drainage, lineament density, elevation, distance from the mainstream, distance from roads, distribution map of precipitation, soil type, geology/hardness, topographic wetness index) to determin the locations of high-risk flash floods and determin potention locations for dam sites. All 11 variables were reclassified into five classes as equal intervals and combined, weighted and ranked for their potential impact on flash floods through the AHP weights (Tables, 1, 2, 3).

**Table 1:** The randomness index (R.I.) of the number of factors (n) used to evaluate consistency^21^.

*N*	1	2	3	4	5	6	7	8	9	10
**RI**	**0**	**0**	**0.58**	**0.90**	**1.12**	**1.24**	**1.32**	**1.41**	**1.45**	**1.49**

**Table 2:** Shows thematic layers, a pair-wise comparison matrix, and consistency confirmation.

Matrix		Slope	LULC	Rainfall	Drainage density	Lineament density	Elevation	Distance to Mainstream	Distance to roads	Normalized principal Eigenvector
		1	2	3	4	5	6	7	8	
Slope	1	1	1	3	5	2	3	3	1	22.63%
LULC	2	1	1	3	1	3	5	3	1	20.33%
Rainfall	3	1/3	1/3	1	3	1	3	1	1	11.84%
Drainage density	4	1/5	1	1/3	1	1	3	3	1	11.03%
Lineament density	5	1/2	1/3	1	1	1	3	2	1	10.48%
Elevation	6	1/3	1/5	1/3	1/3	1/3	1	3	1	6.51%
Distance to Mainstream	7	1/3	1/3	1	1/3	1/2	1/3	1	1	6.05%
Distance to roads	8	1	1	1	1	1	1	1	1	11.12%

**Table 3:** Weight of factors in the flood susceptibility map of the Wadi Araba Basin.

Factors	Classes	Rank	Weight
Slope	0–72.33	22	5
	72.33–144.67		4
	144.67–217.015		3
	217.015–289.35		2
	289.35–361.69		1
LULC	Vegetation	20	5
	Water bodies		5
	Agriculture		4
	Bare land		1
	Buildings		1
Rainfall	0–19.14	12	1
	19.14–26.028		2
	26.028–32.912		3
	32.912–39.79		4
	39.79–46.68		5
Drainage density	0–241.9	12	5
	241.9–459.92		4
	459.92–677.94		3
	677.94–895.95		2
	895.95–1,113.97		1
Lineament density	0–0.169	10	1
	0.169–0.338		2
	0.338–0.507		3
	0.507–0.676		4
	0.676–0.845		5
Elevation	0–293	7	5
	293–590		4
	590–887		3
	887–1,184		2
	1,184–1,481		1
Distance from mainstream	0–0.009877.009877	6	1
	0.009877–0.019754		2
	0.019754–0.029632		3
	0.029632–0.039509		4
	0.039509–0.049386		5
Distance from Roads	0–6378.53.53	11	1
	6378.53–12757.06.53.06		2
	12757.06–19135.59.06.59		3
	19135.59–25514.12.59.12		4
	25514.12–31892.65.12.65		5

### Analytical hierarchy process (AHP) and weighting

The AHP originally presented by^22^, is a practical and extensively utilized multi-criteria decision-making technique. Typically, it is applied to rank variables using pairwise comparisons on the Saaty 1–9 scale with reciprocal judgments to determine the most dominant component^23^. Weights were derived via the eigenvector method and normalized to sum to 1 prior to overlay analysis. Eight flash flood conditioning factors as Slope, LULC, Rainfall, Drainage Density, Lineament Density, Digital Elevation Model, Distance to Main stream and, Distance from roads were taken into consideration in this study to produce the flood-susceptibility map. Combining AHP with GIS offers a straightforward and effective solution to complex problems because it integrates powerful capabilities for processing large amounts of data with methodologies and tools that enable decision-making, visualization, and mapping^24^.

**Fig. 3:**
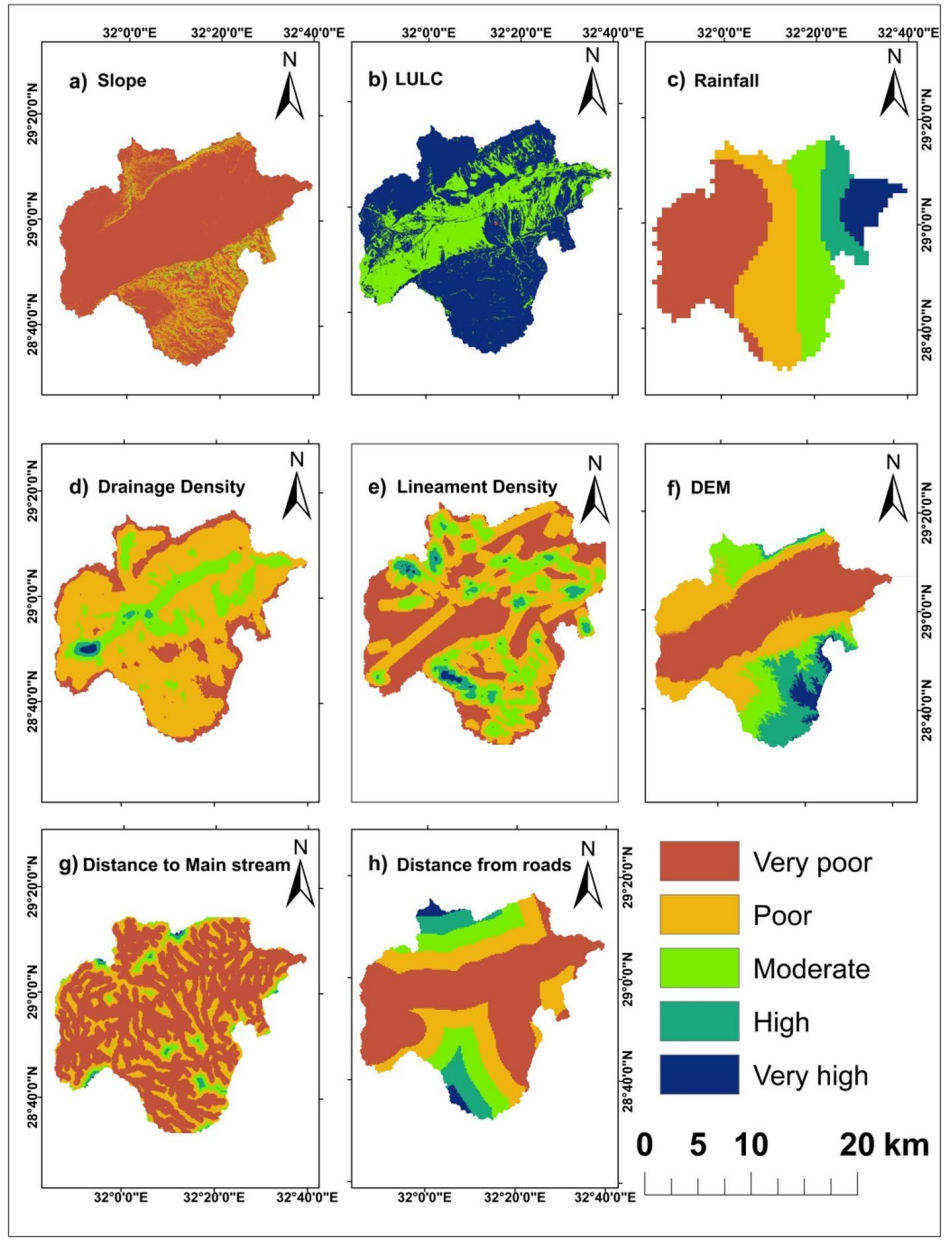
Thematic Layers used in AHP method for Geohazards detection after reclassification (**a**) Slope. (**b**) LULC. (c) Rainfall. (**d**) Drainage Density. (**e**) Lineament Density. (**f**) Digital Elevation Model. (**g**) Distance to Main stream. (**h**) Distance from roads.

**Fig. 4:**
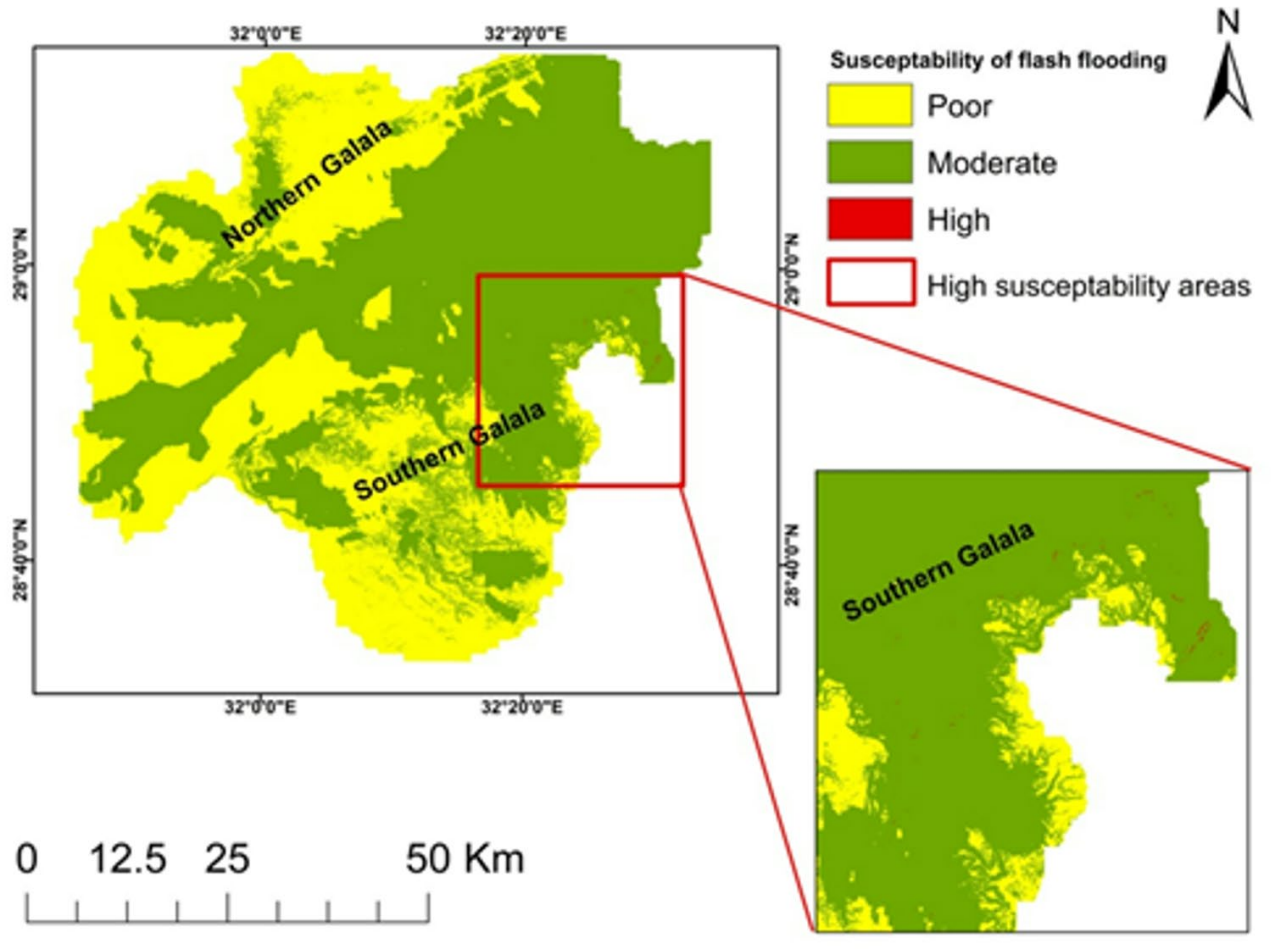
Flash flooding susceptibility maps in Wadi Araba basin.

Rationale for weights and pairwise judgments: Slope and LULC received the highest weights because slope governs runoff acceleration and response time across escarpments, while LULC modulates imperviousness and effective infiltration, intensifying overland flow and exposure in built/barren classes (Table 2). Rainfall was weighted highly as the primary climatic driver of flash-flood generation in hyper-arid settings, whereas drainage density reflects channel connectivity and concentration potential, warranting mid-to-high weight consistent with basin organization (Fig. 3d, 4). Lineament density received a mid-weight in surface susceptibility because fractures may enhance infiltration and partially decouple surface hazard from subsurface pathways, while remaining important for dam siting to discriminate storage (low lineaments) versus recharge (high lineaments) objectives. Distance to roads was assigned a mid-weight as a proxy for exposure and flow concentration along linear infrastructure, while elevation and distance to main stream were given lower weights as secondary controls relative to slope, drainage organization, and land cover in this basin. Pairwise comparisons satisfied Saaty’s consistency (CR = 0.09 < 0.10; *n* = 8, RI = 1.41) for flash flooding susceptibility and (CR = 0.0736 < 0.10; *n* = 10, RI = 1.49) for dam sitting suitability, and the normalized principal eigenvector yielded the final criteria weights reported in Table 2.

#### Pairwise comparison matrix

At this stage, a weight is determined for each parameter based on the degree of its impact on flash floods. The relevant significance or value was determined by using the relative significance scale. The length of the comparison matrix table is related to the number of elements used in the study area. As indicated in Tables 4, 5, 6, 7 and 8, 9 variables weight, classes, and The CR values have been determined for the table of comparison matrix. The following expression calculates the CR value^22,25^. The consistency ratio was computed as Eq. 1. With the acceptance criterion CR ≤ 0.1, the normalized principal eigenvector provided the weights reported in Table 2 (flood-susceptibility and Table 6 (dam siting)

1 $$\:CR=CI/RI$$

**Table 4:** Statistics about flash floods in the region under investigation.

Class	Area (Km^2^)	Percentage (%)
Low	1671.341	41.506681
Moderate	2354.803	58.480043
High	0.5346	0.013276

**Table 5:** Weights and ranks classification for the ten variables.

Factors	Classes	Rank	Weight
Elevation	0–293	10	1
	293–590		2
	590–887		3
	887–1,184		4
	1,184–1,481		5
Slope	0–72.33	15	1
	72.33–144.67		2
	144.67–217.015		3
	217.015–289.35		4
	289.35–361.69		5
Soil type	Limestone and Dolomite	12	1
	Limestone and Sandstone		2
	Sand and Gravel		3
	Shale and Limestone		4
	Sandstone and Limestone		5
Geology	Tertiary	10	1
	cretaceous		2
	Quaternary		3
	Paleozoic		4
	carboniferous		5
Rainfall	0–19.14	4	1
	19.14–26.028		2
	26.028–32.912		3
	32.912–39.79		4
	39.79–46.68		5
Lineament density	0–0.169	5	5
	0.169–0.338		4
	0.338–0.507		3
	0.507–0.676		2
	0.676–0.845		1
Distance to roads	0 − 6378.53	4	5
	6378.53–12757.06		4
	12757.06–19133.59		3
	19133.59–25514.12		2
	25514.12–31892.65		1
Land use/Land cover (LULC)	Vegetation	6	3
	Water bodies		5
	Agriculture		3
	Bare land		3
	Buildings		1
Stream density	0–241.9	14	1
	241.9–459.92		4
	459.92–677.94		5
	677.94–895.95		3
	895.95–1,113.97		2
Topographic Wetness Index (TWI)	−4.6827	20	1
	−4.6827–0.0231		2
	0.0231–4.729		3
	4.729–9.4349		4
	9.4349–14.14078		5

**Table 6:** Illustrates thematic layers, a pairwise comparison matrix, and consistency confirmation.

Elevation	1	2	3	4	5	6	7	8	9	10
	1.00	1.00	1.00	1.00	3.00	3.00	3.00	3.00	0.33	0.33
Slope	1.00	1.00	3.00	3.00	5.00	2.00	3.00	1.00	1.00	0.33
Soil type	1.00	0.33	1.00	3.00	5.00	3.00	2.00	3.00	1.00	0.33
Geology	1.00	0.33	0.33	1.00	3.00	3.00	3.00	1.00	1.00	1.00
Rainfall	0.33	0.20	0.20	0.33	1.00	1.00	2.00	1.00	0.33	0.33
Line density	0.33	0.50	0.33	0.33	1.00	1.00	3.00	1.00	0.33	0.33
Distance to road	0.33	0.33	0.50	0.33	0.50	0.33	1.00	1.00	0.33	0.20
LULC	0.33	1.00	0.33	1.00	1.00	1.00	1.00	1.00	0.33	0.33
Stream density	3.00	1.00	1.00	1.00	3.00	3.00	3.00	3.00	1.00	1.00
TWI	3.00	3.00	3.00	1.00	3.00	3.00	5.00	3.00	1.00	1.00
Sum (col)	11.33	8.7	10.7	12	25.5	20.33	26	18	6.667	5.2
weight	0.115	0.141	0.136	0.102	0.047	0.057	0.034	0.051	0.138	0.18

**Table 7:** Shows the area and percentage of dam locations in the Wadi Araba area.

Suitability	Area	Area (%)
very low	70.33	1.75
low	2351.49	58.43
moderate	1597.50	39.70
high	4.89	0.12

**Table 8:** Showcase the comparative statistics between the three sites chosen for the dam construction.

Contour Line (Elevation)	Dam 1	Dam 2	Dam 3
	140	120	80
length (m)	688	713	836
Height (m)	13	16	8
Volume of Lake (m³)	31609531.25	3490781.25	758281.25

**Table 9:** Shows the categorization of the AUC curve.

Poor	0.5–0.6
Medium	0.6–0.7
Good	0.7–0.8
Very good	0.8–0.9
Excellent	0.9–1.9

#### Consistency index (CI)

One common way to check for consistency is via the maximum-eigenvalue criterion. The following equation is used to calculate the value of *λ* max:

2 $$\:{\lambda}max={\sum\:}_{j=1}^{n}aij\:\frac{wj}{wi}=n$$

While CR is consistency matrix. The matrix is consistent if CR is equal to 0. If CR is greater than 0, the matrix is inconstant to some degree. Utilizing the eigenvalue approach to get the Principal Eigenvalue (λ), it’s possible to compute the consistency ratio (CR) from the Consistency Index (CI) according to the following equation:

3 $$\:\mathbf{C}\mathbf{I}=\:\frac{({\lambda}\mathbf{m}\mathbf{a}\mathbf{x}-\mathbf{n})}{(\mathbf{n}-1)}$$

In this equation, λ max indicates the principal eigenvalue and n is the number of variables.

### Validation methodology

Model performance was assessed using Receiver Operating Characteristic (ROC) analysis and Area under the Curve (AUC) metrics. ROC curves illustrate the relationship between sensitivity (true positive rate) and specificity (false positive rate). AUC values range from 0 to 1, where higher values indicate better model accuracy. AUC interpretation follows standard guidelines: poor (0.5–0.6), medium (0.6–0.7), good (0.7–0.8), very good (0.8–0.9), and excellent (0.9–1.0.9.0).

### Variables utilized in geohazard and dam site assessments

#### Slope

**Fig. 5:**
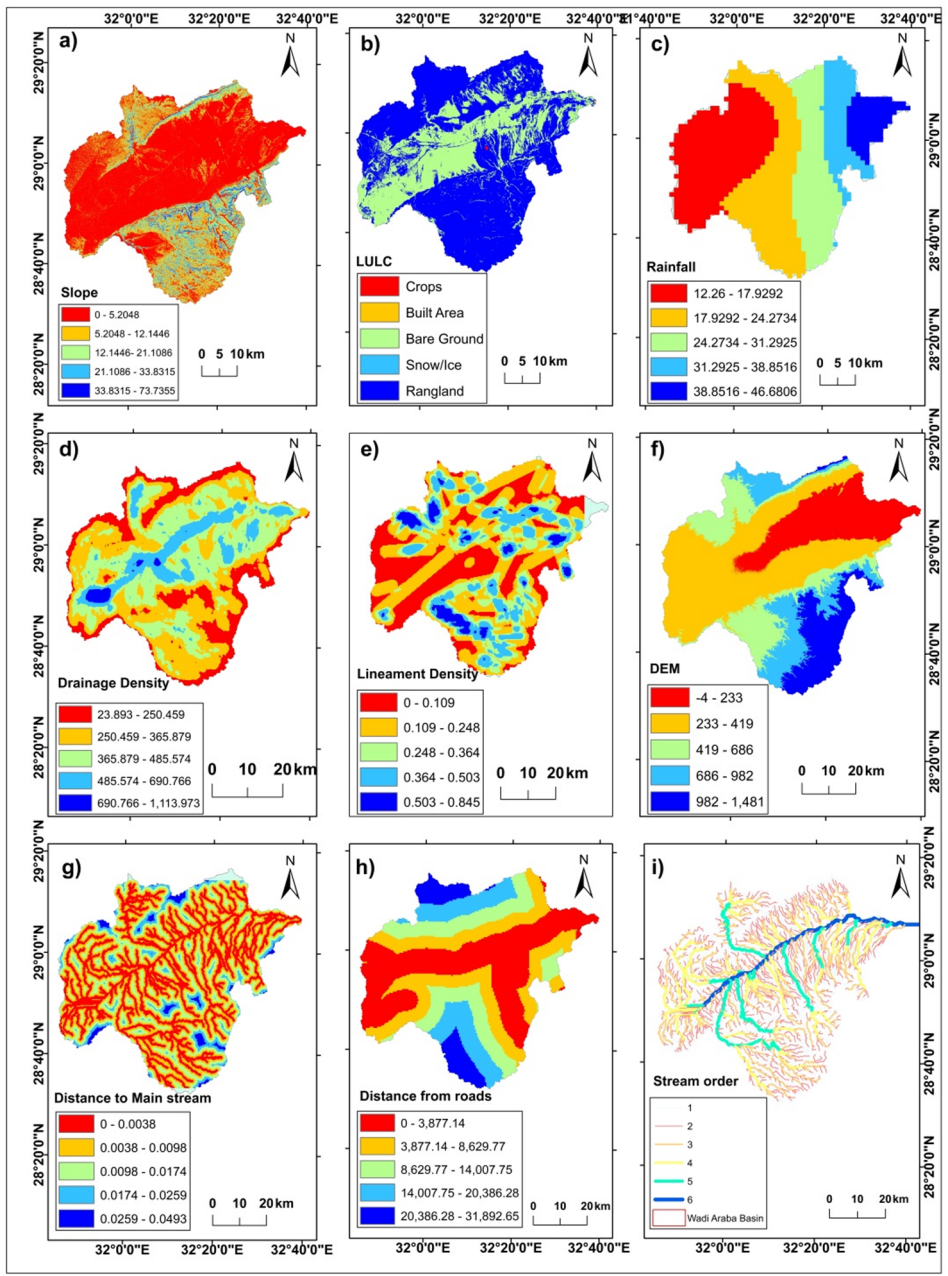
Thematic Layers used in AHP method for Geohazards detection (**a**) Slope. (**b**) Land use/Land cover (LULC). (**c**) Rainfall. (**d**) Drainage Density. (**e**) Lineament Density. (**f**) Digital Elevation Model. (**g**) Distance to Main stream. (**h**) Distance from roads. (**i**) Stream order in the Wadi Araba basin. (All layers were generated and processed using ArcGIS Desktop version 10.8 (Esri, Redlands, CA, USA; https://www.esri.com/arcgis).

The slope is a particularly essential indicator for establishing and identifying flash areas susceptible to flooding since it indicates elevation variation and has a direct influence on watersheds^26,27^, runoff acceleration, and the ability to infiltrate^27,28^. The slope classes with smaller values obtained a higher rating due to flash floods^26^, as lower slope areas are more susceptible to flooding^29–31^. Shallow places are especially susceptible to flooding. Several studies have found positive associations between slope and vulnerability to flooding^32,33^. As a result, there is more water discharged from the streams, causing floods^28^. High slopes lead to increased runoff^34^. Values of slope showed in Fig. 5a.

#### Landuse landcover

Several landuse patterns affect flood intensity and severity^33,35^. The LULC data is a key variable in defining flash-flood locations^36–38^, whereas impermeable surfaces, including barren lands, highways, and residential areas, have been verified to increase rainfall runoff^39^. LULC datasets were gathered from Esri Sentinel-2 Land Cover Explorer applicaion (https://livingatlas.arcgis.com/landcoverexplorer) (Fig. 5b), classes were harmonized to study-specific categories prior to reclassification, and the product’s published accuracy characteristics were considered when interpreting map uncertainty., the spatial distribution of these classes is summarized in Table 3.

#### Rainfall

**Fig. 6:**
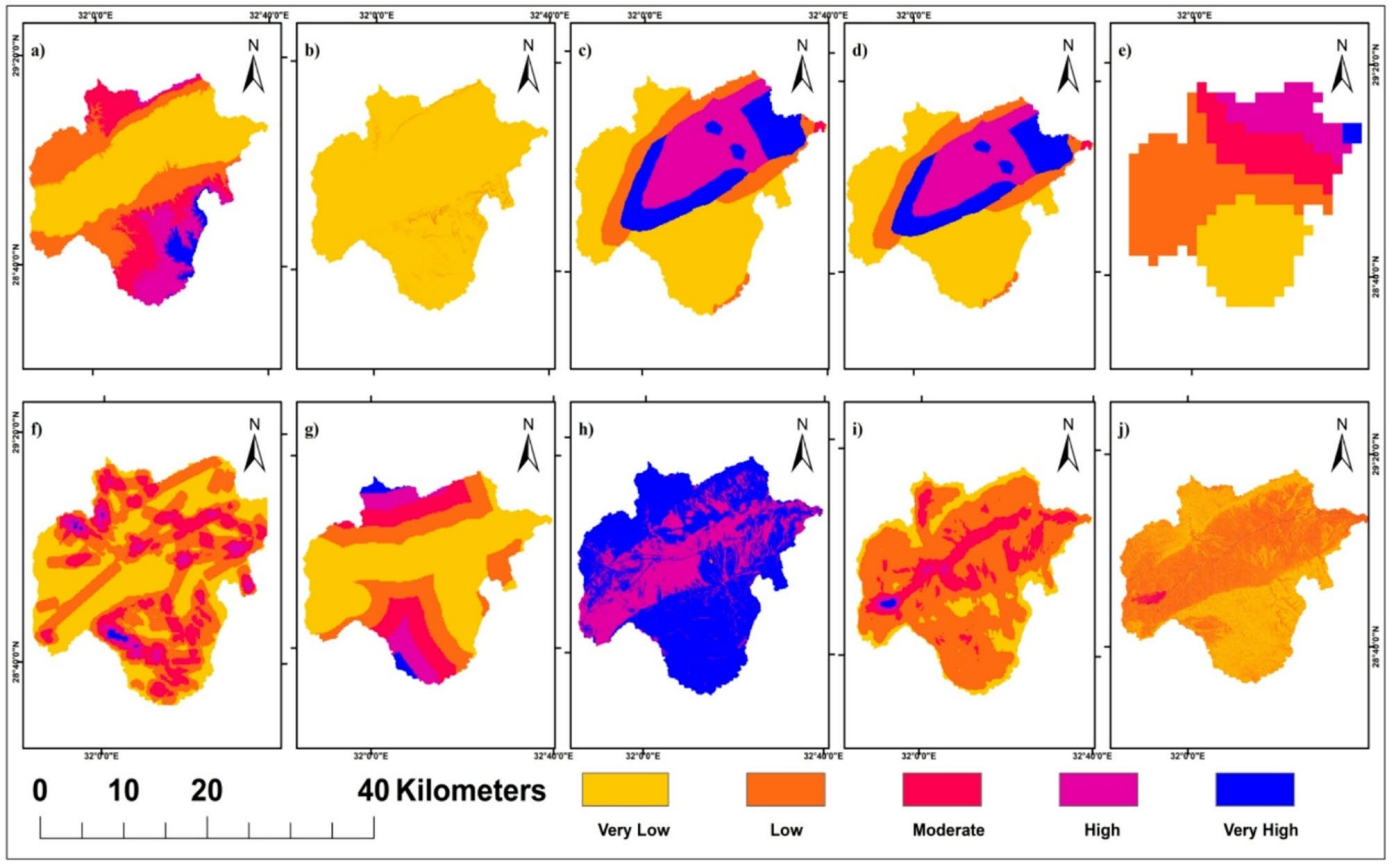
The reclassified thematic layers used to detect the Dam site location in Wadi Araba area. (**a**) Digital Elevation Model. (**b**) Slope of Elevation. (**c**) Soil Texture. (**d**) Geology. (**e**) Rainfall. (**f**) Lineament Density. (**g**) Distance from roads. (**h**) Land use/Land cover (LULC). (**i**) Drainage Density. (**j**) Topographic Wetness Index (TWI). (Generated and processed in ArcGIS 10.8 (https://www.esri.com/en-us/arcgis/products/arcgis-desktop/overview*)*.

Short, intense rainfall can cause flash floods^40^. Precipitation was sourced from the University of East Anglia Climatic Research Unit gridded Time Series (CRU TS Version 4.06), monthly precipitation at 0.5° × 0.5° spatial resolution; specifically, the CRU TS v4.x release (Harris and Jones) accessed via CEDA. Monthly values (2011–2021) were aggregated to annual totals per grid cell and bilinearly resampled to the 30 m analysis grid prior to five-class reclassification. The data from 2011 to 2021 showed that the quantity of rainfall in the region under consideration varied between 12.26 mm/year and 46.69 mm/year, as shown in (Fig. 5c). A substantial quantity of previous studies has proven the link between precipitation and the incidence of floods in an area^41–43^. It is impossible to forecast with precision how much rainfall will cause floods^44^. Alternatively, it is conceivable to argue that, in all conditions, rainfall is the main factor causing flooding^45^Rainfall has been identified by several studies conducted globally as one of the key impacting components for mapping the hazards of flash flooding^46,47^. Annual totals were summarized into five classes (Fig. 3c). Rainfall was divided into five classes: 12.26–17.93 mm/year, 17.93–24.27, 24.27–31.29, 31.29–38.85, and 38.85–46.68 mm/year, as summarized in (Figs. 6).

#### Drainage density

The obtained drainage density values range from 23.893 to 1113.973 (Fig. 2d). High drainage density is associated with rapid runoff response to intense rainfall. Conversely, low drianage density indicates well-joint bedrock with higher infiltration, allowing precipitation to recharge water-bearing formations. The drainage density map was generated from drainage network (Fig. 5d) using the hydrology tools in ArcMap toolbox. Drainage density was computed as the total stream length per unit area (km km⁻²) from a DEM- derived network, using a specified flow- accumulation threshod to delineate channels before converting to polylines for density calculation. It is defined as the ratio of total channel length [L] to watershed area [A] (Fig. 3d). Units are reported as km km⁻².

$${\text{Density of drainage }}\left( {{\mathrm{DD}}} \right)\,=\,{\mathrm{L}}/{\mathrm{A}}$$

#### Lineament density

A lineament is a linear feature that delineates the underlying structures, that delineates the underlying structures, such as cleavages, discontinuity surfaces, and fractures and faults. It expresses linear to slightly curvilinear structural characteristicd that differ from surrounding patterns and reflects certain underlying features^48^. Lineaments were mapped from multi-directional hillshade and directional filtering on the DEM with a minimum segment-length threshold and manual verification against lithologic trends; density was calculated as line length per area (km km⁻²) within a fixed window (Fig. 5e).

#### DEM (Elevation)

The USGS gives download access for DEM using SRTM data (https://earthexplorer.usgs.gov/) that have a spatial resolution of 30 × 30 m and ArcGIS is used to visualize watersheds, stream networks, elevation, slope, drainage density, lineament density, and morphometric data using DEM. Water descends from higher altitudes to lower ones, and low-lying places have a very high chance of flooding^49^. The research area’s elevation ranges from − 4 to 1481 m above sea level as shown in (Fig. 3f). Sinks were filled on the DEM prior to hydrologic modelling to ensure continuous drianage for flow-based derivatives, following standard hydrology toolset practice. The DEM was SRTM 1 arc-second V3, void-filled, at 30 m resolution.

#### Distance to main stream

The distance from land to main streams influences the amount of water in rock and soil^50^. These variables can influence the recharging procedure in comparison with farther distances from the main stream. The recharging process could possibly be affected by it. Closer proximity to waterway systems increases the likelihood of infiltration. The research region’s DEM was used to construct five groups (Fig. 3g). Euclidean distance to the DEM-extracted main stream was computed on the common grid.

#### Distance from roads

According to^51^another crucial consideration when identifying areas that are vulnerable to flash floods is the distance between the road and any location in the study area. Furthermore, because runoff processes accelerate and infiltration is reduced, areas near roads are particularly susceptible to flash flooding. The spatial map of road distance was divided into five classifications in this study: extremely low (0–3877.14 m). Low (3877.14–8629.77 m), moderate (8629.77–14007.75 m), high (14007.75–20386.28 m), and extremely high (20386.28–31892.65 m). road polylines were sourced from a puplic vector dataset (BBBike extracts OpenStreetMap (OSM, Garmin, Shapefile etc.), then Euclidean distance to roads was computed and reclassifies into five classes (Fig. 3h).

#### Soil type

**Fig. 7:**
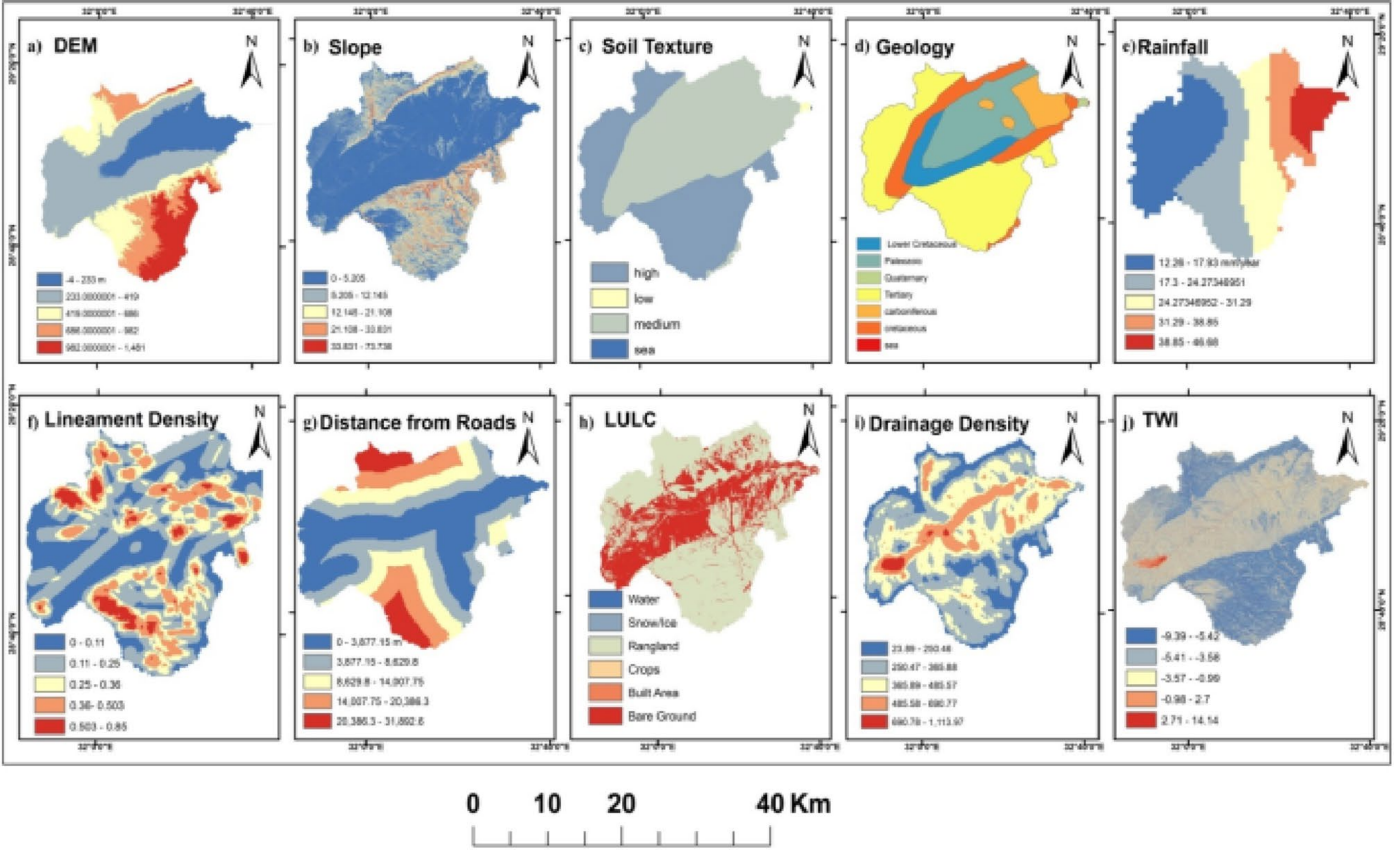
The 10 variables used to detect the Dam site location in Wadi Araba area. (**a**) Digital Elevation Model. (**b**) Slope. (**c**) Soil Texture. (**d**) Geology. (**e**) Rainfall. (**f**) Lineament Density. (**g**) Distance from roads. (**h**) Land use/Land cover (LULC). (**i**) Drainage Density. (**j**) Topographic Wetness Index (TWI). (Generated and processed in ArcGIS 10.8 (https://www.esri.com/en-us/arcgis/products/arcgis-desktop/overview*)*.

Soil texture was downloaded from (https://www.fao.org/) harmonized into five classes reflecting infiltration/runoff behavior and reclassified into five equal-interval bins prior to dam-siting overlay; spatially, limestone dominates western Galala highs, sand–gravel and minor sandstone–limestone occur towards the Gulf of Suez, and shale–limestone with Nubian sandstone blocks characterize the central sector (Fig. 7c).

#### Geology/hardness

Geological formations were grouped into three hardness classes (low/medium/high) to represent foundation competence and construction suitability^41,52^; high-resistance units occur in the SW and NW, medium resistance spans central Lower Cretaceous–Paleozoic–Carboniferous–Cretaceous belts, and low-resistance Quaternary deposits fringe the eastern Gulf of Suez margin (Fig. 7d).

#### Topographic wetness index

TWI was computed to identify saturated zones and moisture- contributing areas within the watershed, then discretized into five equal interval classes for overlay.

#### Dam-siting reclassification

Ten variables were considered for dam site suitability analysis: elevation, slope, soil type, geology, rainfall, lineament density, road distance, LULC, stream density, and TWI (Fig. 7). Soil texture data were classified into five categories (limestone-dolomite, limestone-sandstone, sand-gravel, shale-limestone, sandstone-limestone) based on penetration rates and runoff characteristics. Geological formations were grouped into three hardness classes (low, medium, high) reflecting construction suitability and foundation competence. All layers were reclassified into five equal interval classes before weighted overlay analysis (Fig. 6).

## Results

Flash‑flood susceptibility was mapped using the AHP‑weighted overlay of the selected criteria, and candidate dam locations were delineated accordingly. Both the areas susceptible to flooding and possible dam locations were located using AHP approach and remote sensing data in this study.

### Geohazards assessment model

The final map delineates coherent susceptibility patterns as rare high- risk patches near the southern Galala escarpments, extensive moderate zones along trunk corridors, and low susceptibility across western interfluves (Fig. 4; Tables 4 and 5). Slope values are summarized in Fig. 3a. Large slopes increase the risk of flash floods by facilitating rapid rainfall runoff. In contrast, low slopes present a lower risk. A sudden decrease in slope heightens the flooding risk because it causes a significant volume of water to become stagnant, leading to potential major flooding^28,40,53^. There were five distinct classifications identified on the research area’s slope map. The range of values for the slopes was 0 to 5.2° for the lowest slopes and 33.83 to 73.73° for the highest (Fig. 3a). The lower slope occupying most of the research area, particularly on the Wadi’s floor, while higher slope values were noticed in the northern and southern Galala plateaus.

According to^35^, an area’s Land Use (Fig. 3b) has an essential influence on hydrologic responses at different periods. The study area receives 46.68 mm of rain, which is the maximum amount. The highest rainfall occurs near the Gulf of Suez and decreases gradually with entering the Wadi (Fig. 3c). The density of lineaments in the study area ranges between 0.109 and 0.845 km/km2 as shown in (Figs. 3e and 5e). The drainage appears moderate and high at the center of the basin of Wadi Araba, although it is relatively low in the north and south regions of the basin (Fig. 3d). Elevations are highest on the Galala plateaus and lowest along the Wadi floor. The highest elevation areas are concentrated in the south part of the research area at the Galala Plateau (Fig. 3f).

Distance from Mainstream is an important factor for the sensitivity of flash flooding in the research area. Locations with lower values frequently have more flash floods. On the other hand, areas with higher values are fewer susceptible to flooding. (Fig. 3g).

Flash flooding susceptibility map of the research area depends extensively on the Distance from Roads (Fig. 3h), blocking the rain-water penetration process that can result in a severe flash flood. The study observed that flash flood devastation zones are often found close to the roads. Conversely, higher values of this factor reflect a reduced susceptibility to flash floods.

**Fig. 8:**
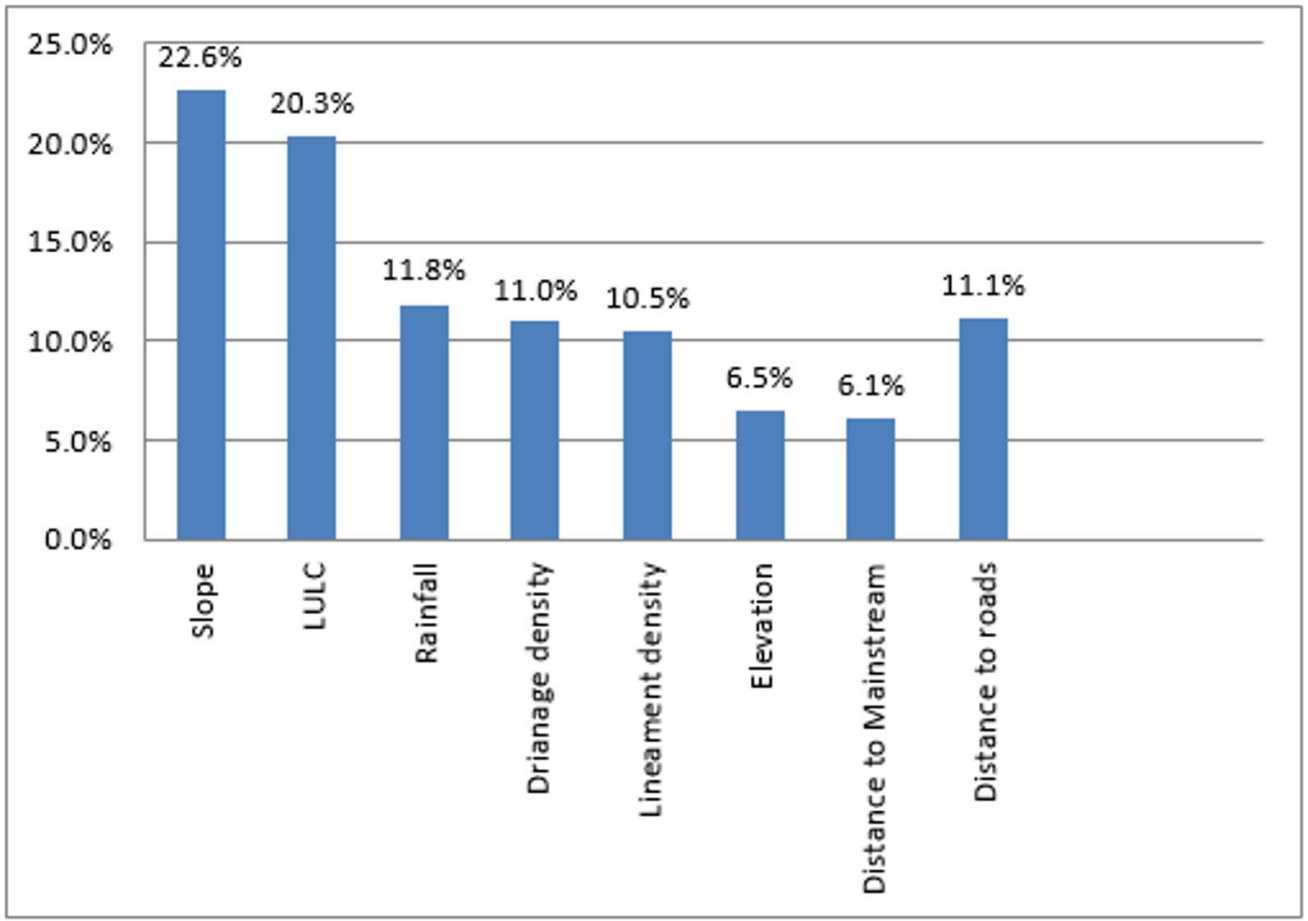
Showcase the thematic layers according to their respective weights that were normalized and assigned to create the Flash Flooding geohazards map.

All of these variables were added to ArcGIS 10.8 software and overlaid to get the final flash flooding map, which illustrates the three classes of the research area: first class is represented as high risk rare sites concentrated in the southeast of the Wadi Araba watershed, especially in the southern Galala plateau, while moderate classes appeared in the center of the Wadi, especially in the area of quaternary deposits, and finally, low in the western part of the Wadi Araba watershed, which indicates low susceptibility to flash flooding shown in Fig. 4, pairwise comparison in Tables 2 and 3; Fig. 8 where the statistics in Table 4. Class areas and proportions are reported in Table 4 (Low = 1671.341 km²; Moderate = 2354.803 km²; High = 0.5346 km²). Validation statistics and interpretation are provided in 4.5 sections.

### Dam site locations

Dam site is a challenging issue since it is influenced by and affects a broad variety of factors, including natural and urban social variables. Furthermore, dams are often created for various reasons, resulting in a more diverse set of variables and impacts.

**Fig. 9:**
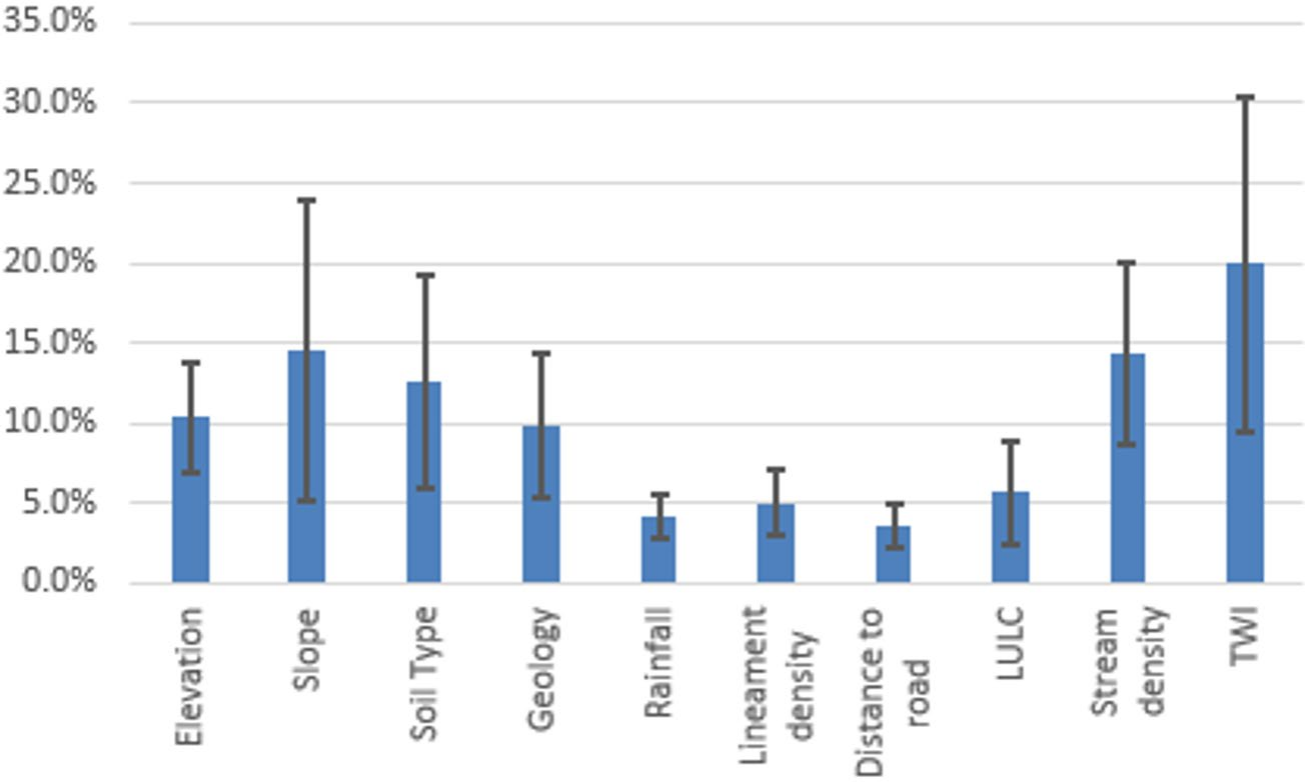
Weights of the normalized thematic layers used to identify expected dam sites.

**Fig. 10:**
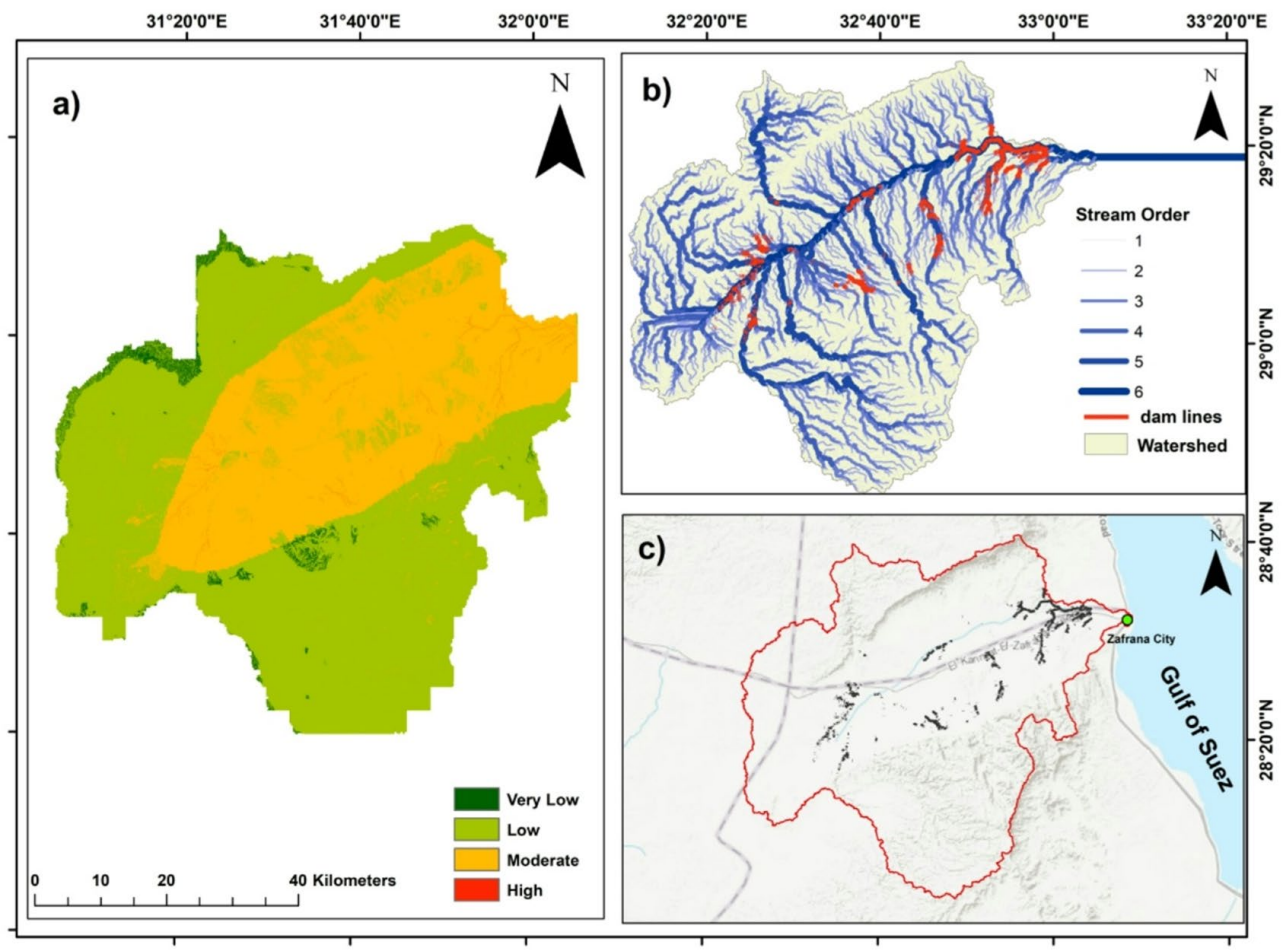
(**a**) Map of dam potentials in Wadi Araba area. (**b**) The high potential areas for building a dam in Wadi Araba watershed illustrated on the drainage pattern of the area. (**c**) The High potential areas for building a dam. (Generated and processed in ArcGIS 10.8 (https://www.esri.com/en-us/arcgis/products/arcgis-desktop/overview*).*

**Fig. 11:**
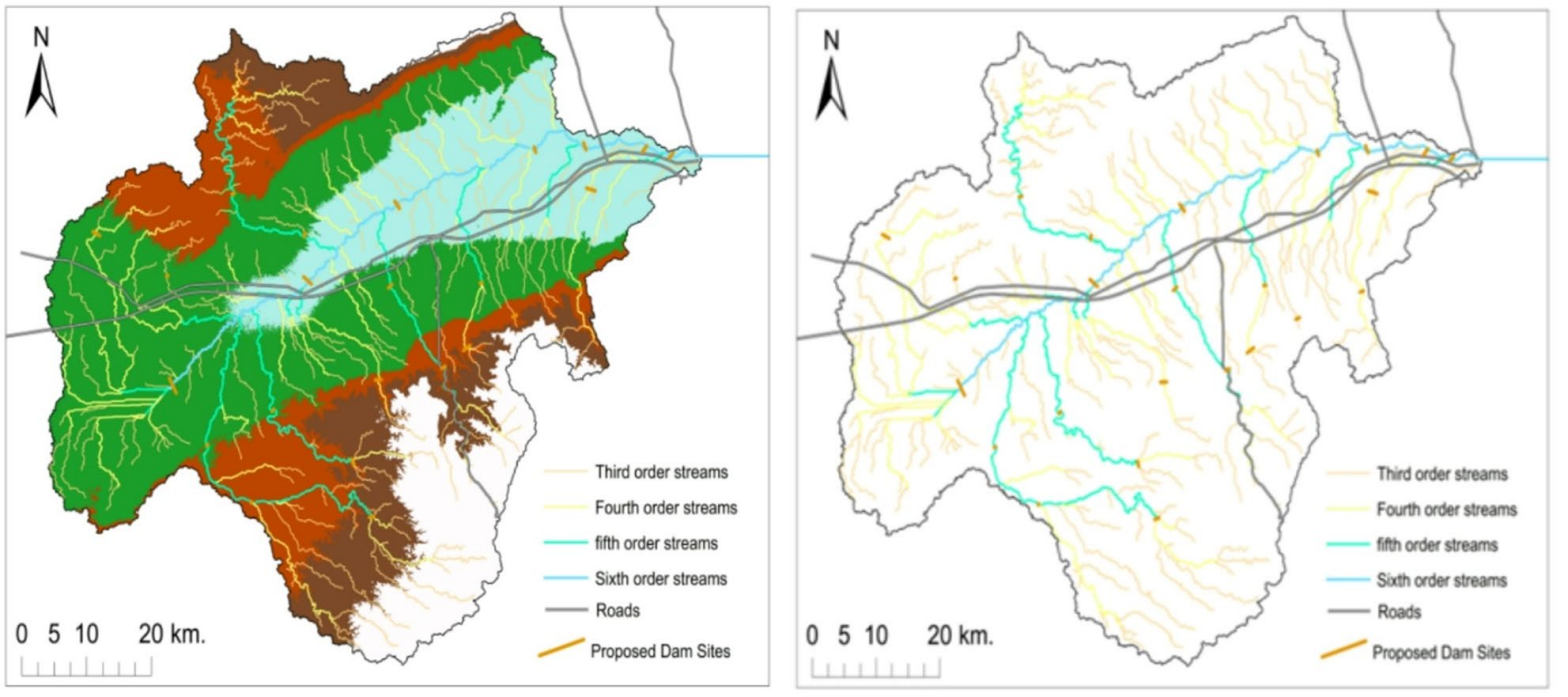
Maps show the suitable sites for building small harvesting dams. (Generated and processed in ArcGIS 10.8 (https://www.esri.com/en-us/arcgis/products/arcgis-desktop/overview*).*

**Fig. 12:**
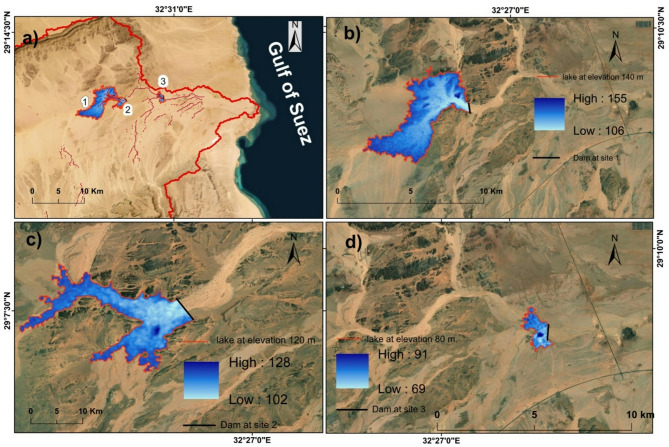
(**a**) the three supposed location for a dam in Wadi Araba basin. (**b**) Dam at location (1) (**c**) Dam at location (2) (**d**) Dam at location (3) (Generated and processed in ArcGIS 10.8 (https://www.esri.com/en-us/arcgis/products/arcgis-desktop/overview*)*.

Dams in the Wadi Araba area were built primarily to prevent flash floods from destroying villages or cities, in addition to enhancing the rate of infiltration in the area, that results in intense underground flow to aid in urbanization and use the under-ground water for supply instead of relying on desalination or even living in desert areas without a continuous source of freshwater. Results for both approaches are summarized in Tables 6, 7 and 8; Figs. 9, 10, 11 and 12.

#### Using AHP technique

Area statistics per suitability class are given in Table 7. Rainfall increases toward the Gulf of Suez by roughly 46 mm/year and coincides with central high-order drainage that governs the spatial pattern of suitability (Figs. 7 and 6). High suitability zones coincide with belts of low lineament density that reduce seepage risk, whereas areas with higher lineament density are ranked lower due to potential leakage from stored water^54,55^(Fig. 7f). Suitability increases at shorter distances to roads due to accessibility and protective value for linear infrastructure; central corridors with denser roads exhibit elevated suitability scores (Fig. 7g).

Built-up areas register low suitability, while bare/gravelly surfaces and active channels dominate moderate-to-high suitability pixels along trunk valleys (Fig. 7h)^56^.

Clusters of higher suitability align with higher stream orders and greater drainage density, where interception of concentrated flows is geomorphically efficient (Fig. 7i)^57^. Mapped TWI highs downstream of valley constrictions co-locate with candidate impoundment reaches, reflecting larger contributing moisture and potential storage footprints^58^, (Fig. 7j).

The composite suitability surface therefore peaks in the central Wadi where higher-order drainage converges, rainfall is relatively higher toward the Gulf margin, distances to roads are shorter, and lineament density is lower, yielding the mapped high-class patches in Fig. 10a.

For the purpose of conducting an exact pair-wise comparison, the value of the CR is compared with 0.1, which is the highest possible CR value^22^. The consistency index (CI) value can be calculated according to Eq. 3, and the consistency ratio (CR) for Table 6 is 0.0736, according to Table 1 and Eq. 1, which is lower than the highest possible CR value that the AHP may recommend. Thus, the consistency of pairwise comparison is accepted.

As the map shows (Fig. 10a), Northwest and southwest small portions showed a very low suitability for constructing dams, and most of the area dropped in the range of low suitability at the northern and southern Galala plateau, and moderate suitability lies within the center of the Wadi Araba area. The high suitability areas for constructing a dam lie within the central area due to the drainage order’s presence, which indicates a high flow of rainwater. In general, 0.12% of the area of Wadi Araba dropped in the range of high suitability. 39.70% of the area dropped in the range of moderate suitability; 58.43% has got low suitability; and 1.75% of the area of Wadi Araba was in very low suitability for the building of dams, as shown in Table 7.

#### Using ArcGIS to build small dams

Small earth dams are rainwater storage structures built across small valleys to capture runoff from upstream catchment regions^59^. Using the drainage network and contour lines for small dams were delineated representing elevation of high stream order that accumulate an excessive amount of water and are at an appropriate elevation for a dam as shown in (Fig. 11). Suggested locations for dams were selected using a combined dataset that included three-dimensional contour lines, a suitability layer, and the drainage ordering layer.

### Dam locations and artificial lakes map

The three sites for the dam that met the criteria were chosen based on the morphological study of the basin, illustrated in Fig. 12a. Additionally, an assessment of the three suggested locations that meet the site needs and requirements was done. As shown in Fig. 12b and c, and d and Table (8) that shows the dam’s height and length with the lake’s volume (site 1 prioritized by storage geometry).

### Profile of suggested dams

**Fig. 13:**
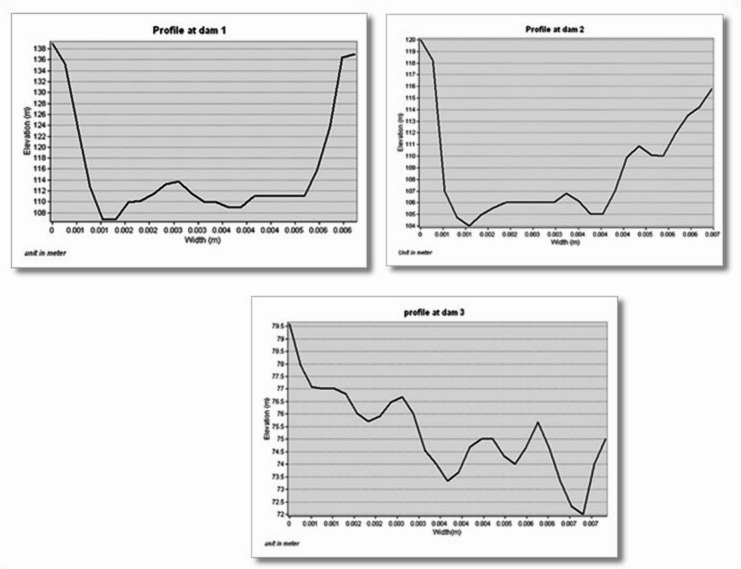
A cross-sectional views of the three different dam sites.

**Fig. 14:**
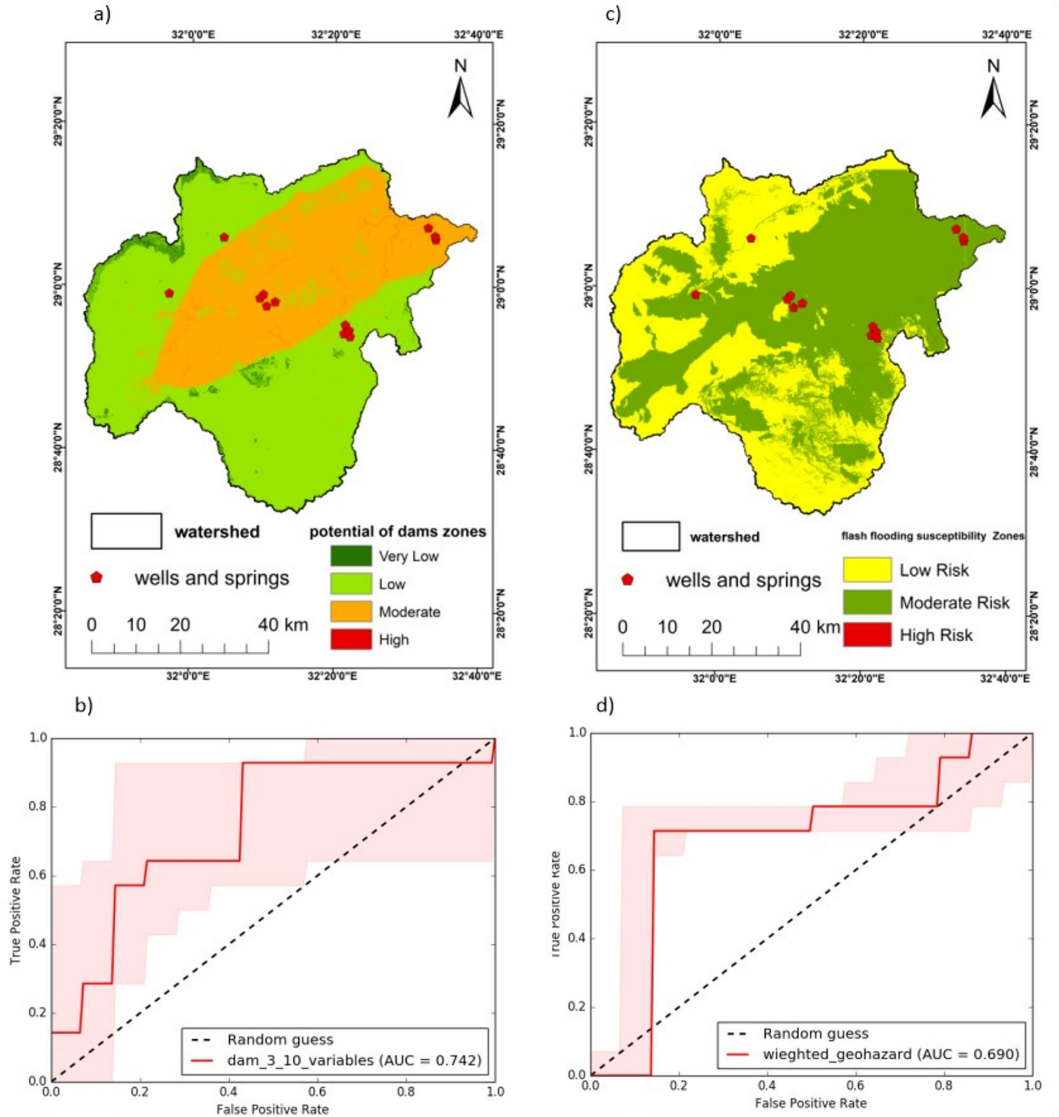
(**a)** suitability map for dam site locations. (**b)** ROC curve for dam site selection and flash flooding in the study area. (**c)** Suitability map for flash flooding areas. (**d)** ROC curve for flash flood susceptibility zonation. (Generated and processed in ArcGIS 10.8 (https://www.esri.com/en-us/arcgis/products/arcgis-desktop/overview*).*

A profile of the proposed dams was created using the DEM and the dams’ locations and contour layer with intervals of 20 m. Subsequently, a link was discovered between the vertical elevations on each of the indicated places and the corresponding horizontal distances that were required in order to produce the lateral shape of the valley stream. To determine the possibility of construction, the dam’s maximum elevation was examined. The highest allowable height for the dam at the first location is 31 m, and 688 m is the greatest horizontal distance, or the dam’s width, that corresponds to this height. As illustrated in (Figs. 13 and 14) and Table (8), the second place has a maximum height of 16 m and a max horizontal distance of 713 m (Fig. 13), and the third dam height is 8 m, resulting in a total length of 836 m (Fig. 13).

Therefore, compared to the second and third locations, the first location’s side sector had a more uniform shape, which would help with dam stability and lower construction costs. According to the findings the first location is the one that is most appropriate for the building of the dam for the reasons that are listed below: It has a larger storage capacity than the second and third dams; the width of the valley is smaller than the second and third dams; and the area flooded with rainwater at the lake’s area is smaller in the second and third dams than the first. Furthermore; the dam’s shape is somewhat more consistent than that of the other two dams. The first dam site has a storage capacity of about 31.6 million m³ of rainwater, while the second and third have 3.49 million m³ and 758,281 m³, respectively, which give preference to the first location also (most suitable).

### Validation

Overall, the AUC value for dam site potential was a good model with 0.7–0.8 value that indicates a good site for building a dam (Fig. 14). The AUC value of the current study for flash flooding geohazards was medium model with 0.6–07 value^60^, (Figs. 14a, b, c, and b) and this showed that the AHP model is suited for recognising flash flood susceptibility zonation for the current research. Observed wells and springs are concentrated along the central of Wadi Araba and the northern Galala plateau, with only a few in the northern Galala, closely tracking the mapped gradients in dam‑suitability and flood‑susceptibility (Fig. 10a, c). The ROC analysis shows acceptable‑to‑good discrimination for the dam‑siting model (AUC = 0.742; panel b) and moderate discrimination for the flood‑susceptibility model (AUC = 0.690; panel d), both clearly above the 0.5 random baseline and therefore informative across thresholds.

## Discussion

This study shows that an integrated RS–GIS and AHP framework can deliver an operational flash flooding susceptibility zonation for the Wadi Araba basin while, in parallel, prioritizing a very limited set of dam site locations suitable for flood mitigation and groundwater recharge under hyper‑arid conditions^22,38,61^. The final flash flooding susceptibility map, produced by reclassification of eight thematic layers into five classes and a weighted overlay using AHP‑derived weights meeting the CR ≤ 0.1 criterion, is dominated by the moderate class (≈ 2354.803 km²), with the low class concentrated in the western Wadi Araba (≈ 1671.341 km²) and a very small high‑risk footprint (≈ 0.5346 km²) near the southern Galala Plateau, consistent with the basin’s stepped relief and drainage organization^15,16,21^. Spatial patterns are physically consistent with controls encoded by slope, drainage density, lineament density, elevation (DEM), distance to main stream, distance from roads, LULC, and rainfall: steep escarpments and benches on the southern Galala accelerate runoff and shorten response times, trunk valleys and downstream basins accumulate discharge where drainage density is higher, and proximity to roads elevates susceptibility by inhibiting infiltration and concentrating surface flow along linear infrastructure^26,51,54^. It is important to highlight that the rainfall data utilized in this study reflects the sum of all the storms that happened in a year, not just one storm. In hyper-arid basins like Wadi Araba, flash floods are mostly caused by short-lived, strong convective storms. Thereby, the resultant susceptibility patterns show relative spatial trends instead of absolute flood magnitudes. The chosen rainfall dataset was utilized to ensure that the data was consistent in this area where there isn’t much data. LULC derived from Sentinel‑2 (Esri Land Cover Explorer, 2023) further modulates responses barren/built surfaces paired with gentle slopes increase susceptibility relative to vegetated or agricultural patches, with interpretation mindful of 10 m classification uncertainty in heterogeneous alluvial–bedrock mosaics^4,39^.

The dam site analysis confirms that high suitability occupies only ≈ 0.12% of the area, underscoring the scarcity of cross‑sections that jointly satisfy geomorphic efficiency, manageable crest length, competent foundation conditions, and favorable contributing areas along higher stream orders in the central part of Wadi Araba^61,62^. Among the three proposed locations, Dam 1 exhibits the most efficient storage geometry (lake volume ~ 31.6 million m³) with a shorter crest length and more uniform side sector, reducing construction complexity and improving prospective stability compared with Dam 2 (~ 3.49 million m³) and Dam 3 (~ 0.758 million m³)^61,62^. The prioritization of dam sites should be regarded as a planning-level suitability evaluation that facilitates early decision-making. Prior to implementation, it is essential to do a comprehensive engineering design, encompassing an examination of sedimentation, seepage, foundation problems, and operational concerns. The analysis distinguishes the functional role of lineament density for storage versus recharge: lower lineament density is preferred for water retention and dam tightness, while higher density zones are more suited for enhancing focused recharge away from the impoundment footprint, which should be reflected in design objectives and seepage assessments^48^. Practically, the RS-GIS outputs support staged construction of small earth dams across higher stream orders and targeted hardening of road corridors to shave hydrograph peaks before flows reach critical infrastructure near the Gulf of Suez coastal plain^19,20,62^.

Methodologically, the workflow adheres to the study’s reproducibility goals: all rasters were projected to WGS84/UTM zone 36 N and resampled to a common 30 m grid, reclassification into five classes was applied to each factor, AHP pairwise comparison matrices yielded normalized principal eigenvectors with acceptable CR (≤ 0.1), and model performance was validated using ROC/AUC on independent flood‑impact evidence with explicit class areas and proportions reported^21,22,38^. The AUC value for dam site potential (~ 0.7–0.8) indicates a good screening model for site prioritization, while the flood geohazards AUC (~ 0.6–0.7) is medium and informative yet improvable; in future iterations, reporting bootstrap confidence intervals and complementing single‑number AUC with threshold‑oriented metrics would enhance interpretability for decision‑making under data‑sparse, hyper‑arid regimes^38^. Given the sensitivity of knowledge‑driven MCDM to discretization and weights, robustness checks using alternative reclassification schemes (e.g., quintiles or expert breaks), ± 10–20% weight perturbations around the principal eigenvector, and spatial cross‑validation are recommended, alongside benchmarking against fuzzy‑AHP or machine learning classifiers as external comparators^26,38^. While the dam site prioritization is based on the relative importance of multiple thematic layers derived through the AHP framework, modest variations in weighting schemes or layer classification thresholds are not expected to substantially alter the dominant priority locations, as these locations are primarily controlled by first-order geomorphic constraints such as valley geometry, contributing area, and stream order, which remain invariant across reasonable weighting scenarios. Consequently, the proposed locations demonstrate a degree of robustness suitable for planning-level decision-making in data-scarce, hyper-arid environments.

Two data‑driven limitations offer a roadmap to strengthen external validity: first, rainfall inputs aggregated to annual totals (2011–2021) under‑resolve sub‑daily convective bursts that dominate flash flooding in hyper‑arid basins; incorporating IDF‑aware or event‑scale rainfall predictors and storm catalogs is likely to improve separability and threshold calibration^41,45^. Second, Euclidean distance to main streams and distance from roads provide consistent proxies, but hydrologically conditioned flow‑path distances and cost‑distance metrics may better capture exposure pathways and access constraints across complex relief for both susceptibility mapping and dam operations planning^58,61,62^. Overall, the integrated RS–GIS/AHP framework, with explicit CR checks and ROC/AUC validation, provides a defensible, replicable evidence base to triage flash flooding susceptibility and to rank optimal retention dam sites in Wadi Araba, with immediate implications for DRR and managed aquifer recharge along the Gulf of Suez margin^6,19,20^. CRU TS precipitation (0.5°) may under-resolve localized convective bursts typical of flash-flooding; interpretations therefore emphasize relative spatial gradients rather than absolute totals. Minor uncertainties also arise from 10 m LULC in heterogeneous alluvial–bedrock mosaics and SRTM-derived slopes on steep escarpments; nonetheless, multi-factor integration and five-class reclassification mitigate these effects.

## Conclusions

This study establishes a reproducible RS–GIS, AHP-driven framework for flash-flood susceptibility zonation and dam-site prioritization tailored to Wadi Araba’s hyper-arid, data-scarce setting, delivering decision-ready layers aligned with development and risk-reduction objectives in the Gulf of Suez corridor. The susceptibility model delineates low (1671.34 km²), moderate (2354.80 km²), and high (0.53 km²) classes, with peak exposure along the southern Galala escarpments and moderate classes tracking low-lying trunk corridors for actionable land-use and infrastructure zoning. The dam-siting analysis integrating terrain, drainage, geology, LULC, rainfall, lineaments, and proximity constraints isolates limited high-suitability zones centrally aligned with major drainage orders and a top-ranked site with superior storage geometry. Held-out ROC/AUC shows medium discrimination for susceptibility (≈ 0.6–0.7) and acceptable discrimination for dam suitability (≈ 0.7–0.8); reporting 95% CIs where available is encouraged to evidence that the lower bound exceeds 0.5. Practical implications include targeted protection of transport corridors, siting small earth-dams to attenuate peaks and enhance recharge, and adopting a transparent weighting/consistency protocol (CR ≤ 0.1) that is auditable and adaptable across adjacent wadis. Limitations (rainfall-product uncertainty, incomplete event inventories, 10 m LULC noise) motivate periodic updates, ancillary ground evidence, and scenario testing of weights and thresholds to sustain performance. These outputs support staged construction of small earth-dams along central high-order reaches and targeted protection of transport corridors, informing DRR and managed aquifer recharge in the Gulf of Suez corridor.

## Data Availability

The original contributions presented in the study are included in the article(https:/[www.frontiersin.org/journals/marine-science/articles/10.3389/fmars.2025.1670000/full](http:/www.frontiersin.org/journals/marine-science/articles/10.3389/fmars.2025.1670000/full)). Further inquiries can be directed to the corresponding author.
